# ERK/c-Jun Recruits Tet1 to Induce *Zta* Expression and Epstein-Barr Virus Reactivation through DNA Demethylation

**DOI:** 10.1038/srep34543

**Published:** 2016-10-06

**Authors:** Wei Zhang, Dongjie Han, Pin Wan, Pan Pan, Yanhua Cao, Yingle Liu, Kailang Wu, Jianguo Wu

**Affiliations:** 1 State Key Laboratory of Virology and College of Life Sciences, Wuhan University, Wuhan 430072, China

## Abstract

DNA demethylation plays an essential role in the reactivation of Epstein-Barr virus (EBV) from latency infection. However, it is unclear how epigenetic modification is initiated in responding to stimuli. Here, we demonstrate that ERK/c-Jun signaling is involved in DNA demethylation of EBV immediate early (IE) gene *Zta* in response to 12-O-Tetradecanoylphorbol-13-acetate (TPA) stimulation. Remarkably, Ser73 phosphorylation of c-Jun facilitates *Zta* promoter demethylation and EBV reactivation, whereas knockdown of c-Jun attenuates *Zta* demethylation and viral reactivation. More importantly, we reveal for the first time that c-Jun interacts with DNA dioxygenase Tet1 and facilitates Tet1 to bind to *Zta* promoter. The binding of c-Jun and Tet1 to *Zta* enhances promoter demethylation, resulting in the activation of *Zta*, the stimulation of *BHRF1* (a lytic early gene) and *gp350/220* (a lytic late gene), and ultimately the reactivation of EBV. Knockdown of *Tet1* attenuates TPA-induced *Zta* demethylation and EBV reactivation. Thus, TPA activates ERK/c-Jun signaling, which subsequently facilitates Tet1 to bind to *Zta* promoter, leading to DNA demethylation, gene expression, and EBV reactivation. This study reveals important roles of ERK/c-Jun signaling and Tet1 dioxygenase in epigenetic modification, and provides new insights into the mechanism underlying the regulation of virus latent and lytic infection.

Epstein-Barr virus (EBV) is a human herpes virus and is etiologically linked to a number of diseases, including multiple sclerosis, infectious mononucleosis, nasopharyngeal carcinoma, Hodgkin’s lymphoma, Burkitt’s lymphoma and post-transplant lymphoma^1^,^2^,^3^,^4^,^5^. EBV belongs to the *γ*-Herpes virus subfamily and can establish lifelong latency in the host B cells after primary infection, but readily reactivated to produce infectious immortalizing virus in a small percentage of cells^6^. The switch of EBV from latency to lytic cycle is initiated by the expression of the viral immediate-early gene *Zta*, also known as *BZLF1*, *ZEBRA* and *EB1*^7^,^8^. Zta protein is a transcriptional activator that shares partial amino acid homology to a basic leucine zipper family of transcriptional factors, including c-Jun and c-Fos^9^. The *Zta* gene expression is regulated at the transcriptional level, which alone leads to trigger EBV reactivation cascade^10^,^11^. However, the precise molecular mechanism underlying the regulation of *Zta* gene is largely unknown.

Extracellular signal-related kinase (ERK) pathway plays an essential role in the regulation of Zta expression and EBV reactivation in response to 12-O-tetradecanoylphorbol-13-acetate (TPA) or other reagents^12^,^13^. The c-Jun directly binds on the *Zta* promoter and activates its activity^14^,^15^,^16^,^17^,^18^, and the levels of Zta and phosphorylated c-Jun are much higher in EBV-infected AGS gastric carcinoma cells than in EBV-infected HeLa cells^19^. It is not clearly whether ERK/c-Jun signaling is required for the epigenetic modification, especially DNA demethylation, to trigger Zta expression and EBV reactivation.

DNA methylation in promoter or enhancer regions normally leads to chromatin compaction that serves as a transcriptional silencer to repress gene expression^20^,^21^,^22^, and conversely, DNA demethylation generates a transcriptional active state. Cytosine 5C position methylation is mediated by several different methyltransferase and usually occurs at CpG dinucleotides^23^. Recent studies indicate that the ten eleven translocation (Tet) family proteins are capable of oxidation of 5-methylcytosine (5mC) to 5-hydroxymethylcytosine (5hmC) to erase existing methylation marks by activating DNA demethylation^24^,^25^,^26^. Furthermore, Tet proteins convert 5mC to another two important cytosine derivatives, 5-formylcytosine (5fC) and 5-carboxylcytosine (5caC), in an enzymatic activity-dependent manner^27^,^28^. 5caC is specifically recognized and excised by thymine-DNA glycosylase and replaced by unmodified cytosine^29^. The EBV genome is heavily methylated in latently infected cells and the constitutive activity of lytic viral genes is repressed by methylation, allowing the virus to establish latency infection^9^,^21^,^30^,^31^,^32^. In contrast, the viral genome is hypomethylated during the lytic infection^2^,^21^,^33^. However, the mechanism of demethylation and the role of Tet in EBV reactivation remain largely unknown.

In this study, we demonstrated that TPA-induced modulation of ERK signaling in conjunction with Tet1-mediated demethylation of *Zta* promoter dynamically regulate EBV reactivation. ERK-dependent activation of c-Jun results in the recruitment of Tet1 to the *Zta* promoter to facilitate *Zta* promoter hypomethylation, *Zta* gene expression, and eventually EBV reactivation.

## Results

### ERK/c-Jun pathway is involved in TPA-stimulated demethylation of *Zta* promoter

**Figure 1:**
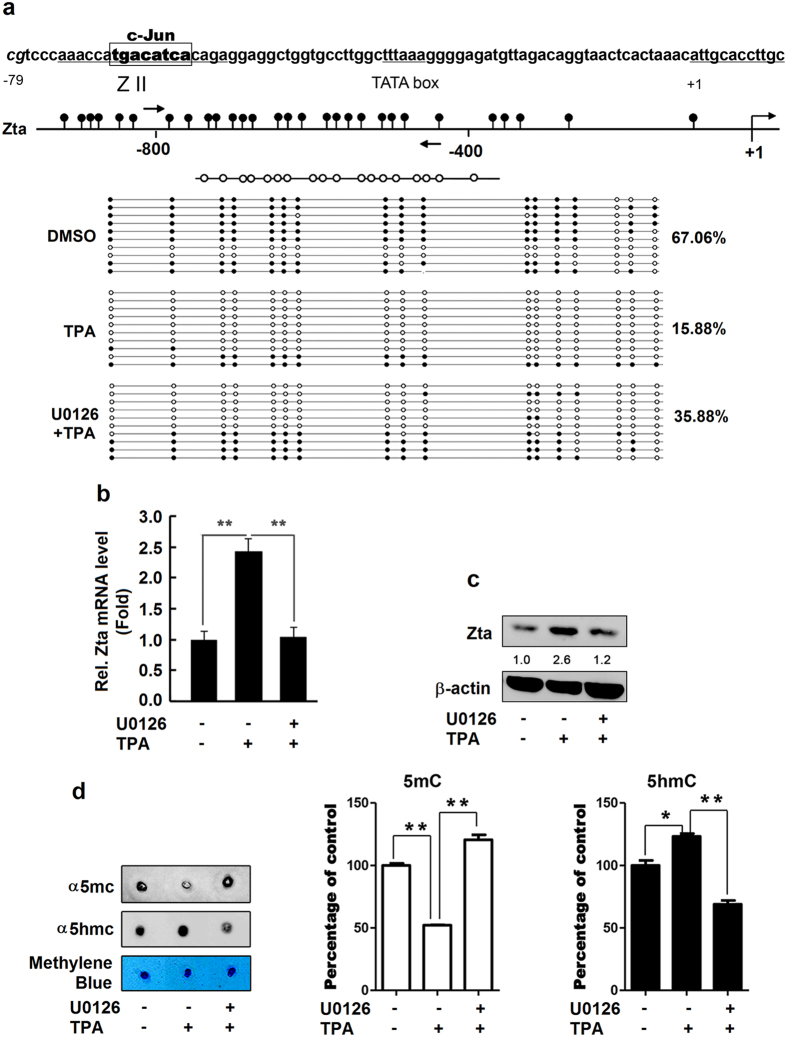
ERK/c-Jun pathway is involved in the demethylation of *Zta* promoter and the expression of Zta gene. **(a**–**c)** B95-8 cells were pretreated with ERK signal pathway specific inhibitor U0126 (20 μM) for 2 h and then treated with phorbol ester 12-O-Tetradecanoylphorbol 13-acetate (TPA) (60 ng/ml) for 24 h. The Zta promoter was analyzed by sodium bisulphite sequencing. The white and black circles indicate unmethylated and methylated CpGs, respectively (**a**). The level of EBV IE gene *Zta* mRNA was determined by real-time PCR (**b**). The level of Zta protein was detected by Western blot analyses (**c**). Relative protein levels, indicated by numbers below the blots, were determined by densitometry, with internal normalization to β–actin and external normalization to the negative control sample processed in parallel. (**d**) B95-8 cells were pretreated with U0126 (20 μM) for 2 h and then treated with TPA (60 ng/ml) for 24 h. The levels of 5-methylcytosine (5mC) and 5-hydroxymethylcytosine (5hmC) were determined by dot blot analyses. Bulk genomic DNA was isolated, sonicated, cross-linked to nitrocellulose membrane, and probed with the monoclonal antibody specific for 5hmC or 5mC. The loading control is showing by the methylene blue stain in the bottom panel. The blot was quantitated by densitometry, and a representative experiment is shown from three independent experiments. All data were collected from 3 independent experiments. *P < 0.05, **P < 0.01 and ***P < 0.001.

The *γ*-herpes viruses immediate early (IE) gene promoter contains a CpG island within 1kb of the transcription start site, which is highly methylated in many epithelial, NK- and B-cell original cancer cell lines and induced promoter demethylation following treatment with TPA and DNA methyltransferase inhibitor azacytidine^2^,^34^,^35^,^36^. However, the mechanism underlying TPA induces epigenetic modification or DNA demethylation is still unknown. As MAPK/ERK signal pathway plays an essential role in expression of *Zta* and EBV reactivation stimulated by TPA^12^,^13^, we speculated that MAPK/ERK signaling may be involvement in the regulation of DNA demethylation. To verify this speculation, B95-8 cells were pretreated with ERK specific inhibitor U0126 and then with TPA and the methylation status of the *Zta* promoter was assessed by sodium bisulphite sequencing. The *Zta* promoter methylation was significantly reduced by TPA (67.06% *vs.* 15.88%), but TPA-mediated repression was largely recovered in the presence of U0126 (15.88% *vs.* 35.88%) (Fig. 1a), indicating that TPA facilitates *Zta* promoter demethylation and ERK pathway is involved in such regulation. Furthermore, demethylation was detected within 1 hour after TPA treatment (Fig. S1). The *Zta* mRNA (Fig. 1b) and Zta protein (Fig. 1c) were stimulated by TPA, but TPA-mediated activations were attenuated by U0126, suggesting that TPA stimulates *Zta* gene expression through ERK pathway. To further validate the effect of ERK on *Zta* promoter demethylation, we examined the production of 5mC and 5hmC in the presence of TPA and/or U0126. Dot blot analyses indicated that 5mC was reduced by TPA and the reduction was recovered by U0126, whereas 5hmC was enhanced by TPA and the enhancement was repressed by U0126 (Fig. 1d), implicating that TPA-promoted DNA demethylation is dependent on ERK activation. Taken together, we demonstrated that TPA stimulates *Zta* promoter demethylation to activate *Zta* expression through ERK pathway.

### Ser73 phosphorylation of c-Jun is essential for ERK-dependent activation of *Zta*

**Figure 2:**
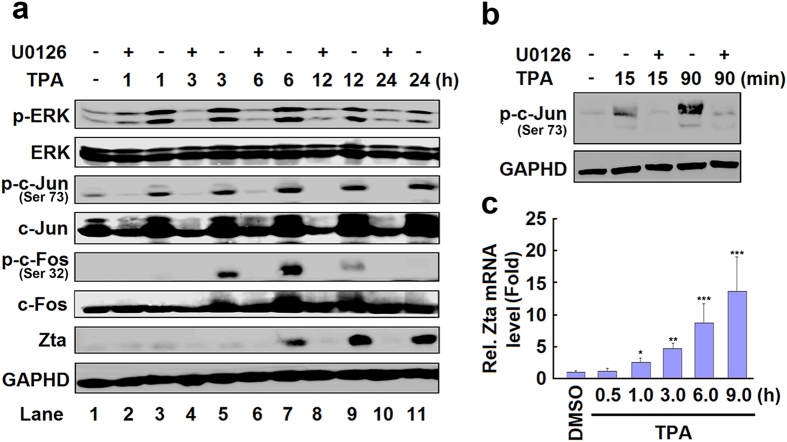
c-Jun Ser73 phosphorylation is essential for ERK-dependent demethylation and activation of Zta promoter. (**a**) B95-8 cells were pretreated with U0126 (20 μM) for 2 h and then treated with TPA for 1, 3, 6, 12, and 24 h, respectively. Whole-cell lysates were isolated and the levels of phosphorylated ERK (p-ERK), phosphorylated c-Jun (p-c-Jun), phosphorylated c-Fos (p-c-Fos), ERK, c-Jun, c-Fos, Zta, and GAPDH proteins were determined by Western blotting using corresponding antibodies. (**b**) B95-8 cells were pretreated with U0126 (20 μM) for 2 h and then treated with TPA for 15 and 90 min. Whole-cell lysates were isolated and the levels of phosphorylated c-Jun (p-c-Jun) and GAPDH proteins were determined by Western blotting. (**c**) B95-8 cells were treated with TPA for 0.5, 1, 3, 6 and 9 h. Total RNAs were isolated from the cells and the level of *Zta* mRNA was analyzed by qPCR. *P < 0.05, **P < 0.01 and ***P < 0.001.

To reveal the mechanism by which ERK signaling stimulates DNA demethylation of *Zta* promoter, the roles of the downstream targets of ERK in TPA-induced *Zta* expression was investigated. Since cellular transcription factors including c-Jun and c-Fos are known to activate *Zta* promoter^14^,^15^,^16^,^18^,^19^,^37^, we examined the involvement of c-Jun and c-Fos in the demethylation of *Zta* promoter. B95-8 cells were treated with MAPK/ERK inhibitor U0126 for 2 h and then treated with TPA for different times. Zta protein production was stimulated by TPA at 6 h and maintained at a high level until 24 h, ERK phosphorylation was induced by TPA from 1 h to 12 h, c-Jun phosphorylation was induced by TPA at 1 h and increased in a time-dependent manner, whereas c-Fos phosphorylation was up-regulated by TPA from 3 h to 12 h, however, TPA-induced production of Zta and phosphorylation of ERK, c-Jun, and c-Fos were repressed by U0126 (Fig. 2a). In addition, Ser73 phosphorylation of c-Jun was stimulated rapidly by TPA within 15 min and further facilitated at a higher level at 90 min, but such activation was attenuated by U0126 (Fig. 2b). Furthermore, *Zta* mRNA was induced by TPA, before c-Fos phosphorylation and after c-Jun Ser73 phosphorylation, at 1 h post-treatment in a time-dependent manner (Fig. 2c). These results demonstrated that c-Jun phosphorylation is occurred before *Zta* expression and suggesting that c-Jun Ser73 phosphorylation is required for *Zta* expression.

### c-Jun is required for ERK-dependent DNA demethylation

**Figure 3:**
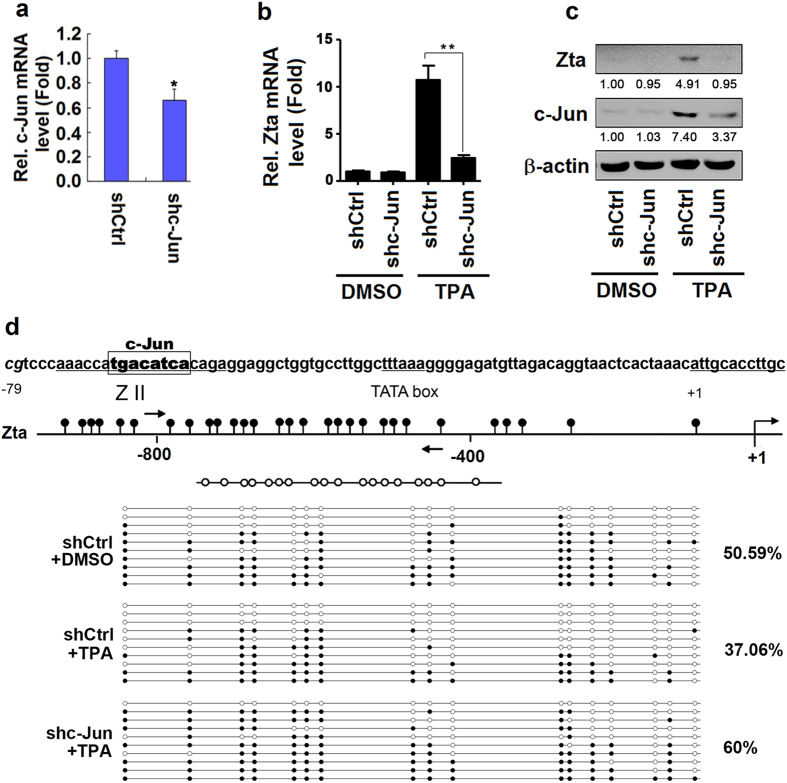
c-Jun is required for ERK-dependent DNA demethylation of *Zta* promoter. (**a**) B95-8 cells were lentiviruses expressing control shRNA (shCtrl) or shRNA against c-Jun (shc-Jun) for 2 weeks and then selected with puromycin. The total RNAs were isolated and the *c-Jun* mRNA was analyzed by qPCR (**a**). (**b**–**d**) B95-8 cells were transduced with lentiviruses expressing shCtrl or shc-Jun for 2 weeks, selected with puromycin, and then treated with TPA for 18 h. The total RNAs were isolated form the cells and the level of Zta mRNA was determined by qPCR (**b**). The cell extracts were prepared and the level of Zta, c-Jun, and β-actin proteins were detected by Western blot analyses (**c**). The *Zta* promoter was analyzed by sodium bisulphite sequencing. The white and black circles indicate unmethylated and methylated CpGs, respectively (**d**). All data were collected from 3 independent experiments. *P < 0.05, **P < 0.01 and ***P < 0.001.

To confirm the requirement of c-Jun for ERK-dependent DNA demethylation induced by TPA, B95-8 cells were transduced with lentivirus expressing shRNA against c-Jun (shc-Jun) for 2 weeks, selected with puromycin, and then treated with TPA. c-Jun mRNA was attenuated in the presence of shc-Jun (Fig. 3a), confirming that shc-Jun is effective. In addition, *Zta* mRNA (Fig. 3b) and Zta protein (Fig. 3c) were activated by TPA, but the activations were attenuated by shc-Jun, suggesting c-Jun is required for TPA-induced expression of Zta. Moreover, the methylation of *Zta* promoter was down-regulated by TPA (50.59% *vs.* 37.06%), whereas this reduction was recovered by shc-Jun (37.06% *vs.* 60.00%) (Fig. 3d), indicating that c-Jun is involved in TPA-mediated demethylation of *Zta* promoter. Taken together, we demonstrated that c-Jun is required for the demethylation of *Zta* promoter induced by TPA.

### c-Jun interacts with Tet1 and recruits Tet1 to the *Zta* promoter

c-Jun is an important transcription factor involved in cell proliferation, transformation, death, somatic cell reprogramming, and lymphoid leukemia^38^,^39^,^40^,^41^. However, there is no evidence to support that c-Jun is directly participated in the modification of methylcytosine. Since we showed that 5hmC level was increased when treated with TPA (Fig. 1d), we speculated that c-Jun may promote *Zta* promoter DNA demethylation through recruiting methylcytosine hydroxylation enzymes (Tet-family proteins).

**Figure 4:**
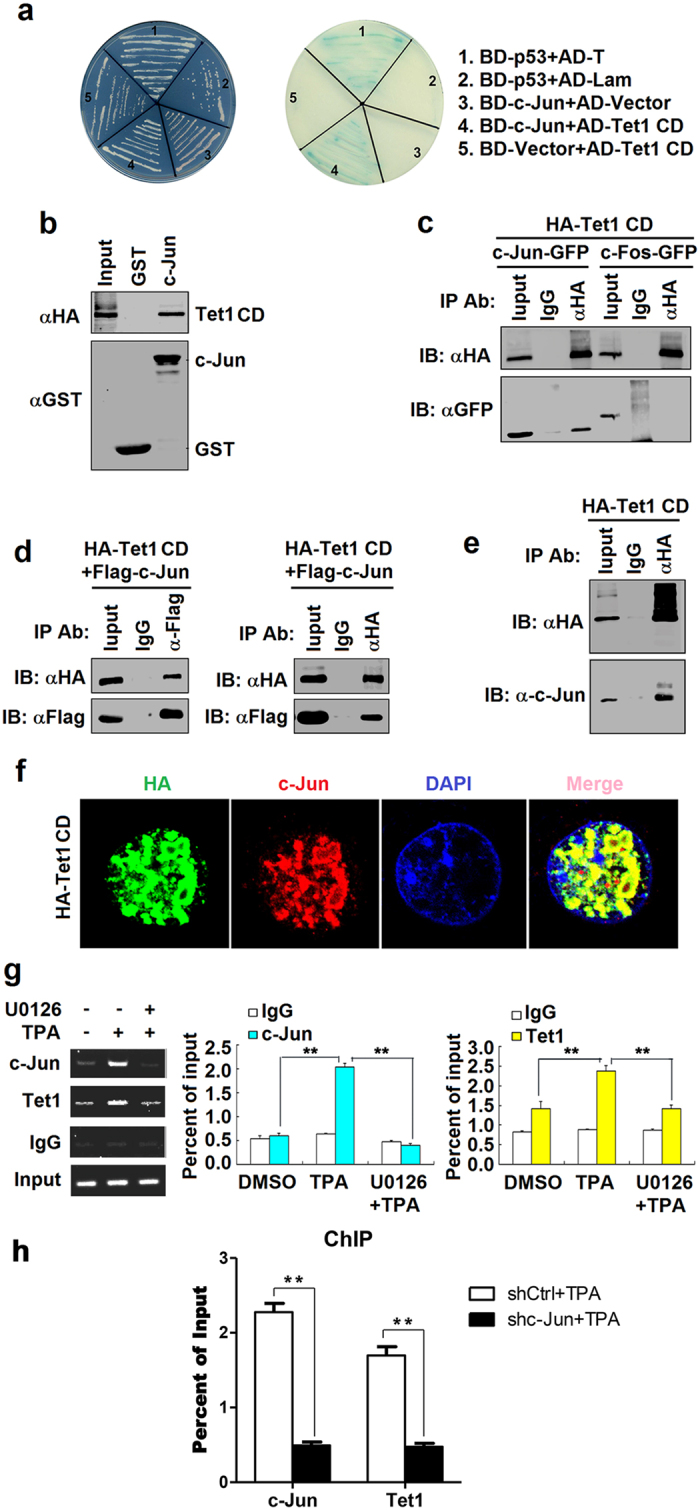
Tet1 binds to *Zta* promoter through interacting with c-Jun. **(a)** Yeast strain AH109 was co-transformed with the combination of one BD and one AD plasmid, as indicated. Transformed yeast cells containing both plasmids were grown on SD-minus Trp/Leu plates (DDO), and colonies were then replica-plated onto SD-minus Trp/Leu/Ade/His plates (QDO) containing X-α-gal to check for the expression of reporter gene (blue colonies). **(b)** GST pull-down assay with GST-c-Jun or GST alone incubated with HA-tagged Tet1CD. **(c)** Co-immunoprecipitation of HA-tagged Tet1 CD and GFP-tagged c-Jun or GFP-tagged c-Fos, followed by western blot analysis with anti-GFP antibody. **(d)** HA-tagged Tet1 CD and Flag-tagged c-Jun were transfected to HEK293 cells and immunoprecipitated using anti-Flag/HA antibody from cell extracts. Co-immunoprecipitation of HA-Tet1/Flag-c-Jun was detected by western blotting using anti-HA/Flag antibody. **(e)** HEK293 cells were transfected with pHA-tagged Tet1 CD and immunoprecipitated using anti-HA antibody from cell extracts. Co-immunoprecipitation of HA-Tet1/ c-Jun was detected by western blotting using anti-HA/ c-Jun antibody. **(f)** HEK293 cells were transfected with HA-Tet1 CD for 24 h. After fixation, the cells were immunostained with anti-HA and anti-c-Jun antibody. The nucleus stained by DAPI and visualized by confocal laser microscopy. **(g)** For ChIP, B95-8 cells were pretreated with U0126 (20 μM) for 2 h and treated with TPA (60 ng/ml) for 4 h. Cross-linked chromatin was immunoprecipitated with the specific antibodies. **(h)** For ChIP, c-Jun knockdown and control B95-8 cells were treated with TPA (60 ng/ml) for 4 h. Cross-linked chromatin was immunoprecipitated with the specific antibodies. Error bars indicate standard error of the mean. *P < 0.05, **P < 0.01 and ***P < 0.001.

To test this hypothesis, we initially performed yeast two-hybrid analyses to identify the interaction between c-Jun and Tet1 (a hydroxylase that binds to DNA and modulates gene methylation and transcription *via* hydroxylation of 5-methylcytosine). Likes BD-p53 interacting with AD-T (as a positive control), a positive interaction was identified between BD-c-Jun and AD-Tet1 CD, but positive interaction was not occurred between BD-c-Jun and AD-vector, between BD-vector and AD-Tet1 CD, or between BD-p53 and AD-Lam (Fig. 4a). To determine whether the interaction of c-Jun with Tet1 is direct, we carried on GST pull-down assays. The results showed that HA-tagged Tet1 CD interacts with GST-c-Jun (Fig. 4b), confirming that c-Jun could bind directly with Tet1. To further examine the interaction of c-Jun and Tet1, co-immunoprecipitation (co-IP) assays were performed, which revealed that GFP-tagged c-Jun, but not GFP-tagged c-Fos, was immunoprecipitated with HA-tagged Tet1 CD (Fig. 4c). Flag-tagged c-Jun was co-immunoprecipitated with HA-tagged Tet1 CD (Fig. 4d, left panel), and reversely HA-tagged Tet1 CD was co-immunoprecipitated with Flag-tagged c-Jun (Fig. 4d, right panel). In addition, the interaction of endogenous expressed c-Jun with HA-tagged Tet1 CD was detected by co-IP assays in HEK293 cells (Fig. 4e). We were also able to coimmunoprecipitate endogenous Tet1 and c-Jun from whole-cell extracts using c-Jun specific antibodies from both control and TPA-treated cells (Fig. S2).

Moreover, immunofluorescence analysis showed that c-Jun was co-localized with HA-Tet1 CD in the nucleus of HEK293 cells (Fig. 4f).

Finally, to determine whether c-Jun and Tet1 are occupied at coincident *Zta* promoter in response to TPA stimulation, we performed chromatin immunoprecipitation (ChIP) assays. As shown in Fig. 4g, we observed much more binding of c-Jun to the *Zta* promoter in response to TPA, more Tet1 was also found to bind to this promoter. Next, we tested whether c-Jun is required for Tet1 binding to the *Zta* promoter in response to TPA using the Erk signal pathway specific inhibitor U0126 (Fig. 4g) or knocking down c-Jun (Fig. 4h). Both U0126 pretreatment and c-Jun knockdown resulted, as expected, in reduced c-Jun activation and binding. More importantly, the decrease in c-Jun occupancy at the promoter coincides with a significant reduction of Tet1 binding to *Zta* (Fig. 4h), demonstrating that Tet1 is recruited to the *Zta* promoter in response to TPA depending on c-Jun. Taken together, we demonstrated that c-Jun interacts with Tet1 and recruits Tet1 to the *Zta* promoter and suggested that ERK/c-Jun signaling is necessary for the binding of Tet1 to TPA-target *Zta* promoter.

### Tet1 is required for ERK-dependent *Zta* promoter demethylation and gene expression

**Figure 5:**
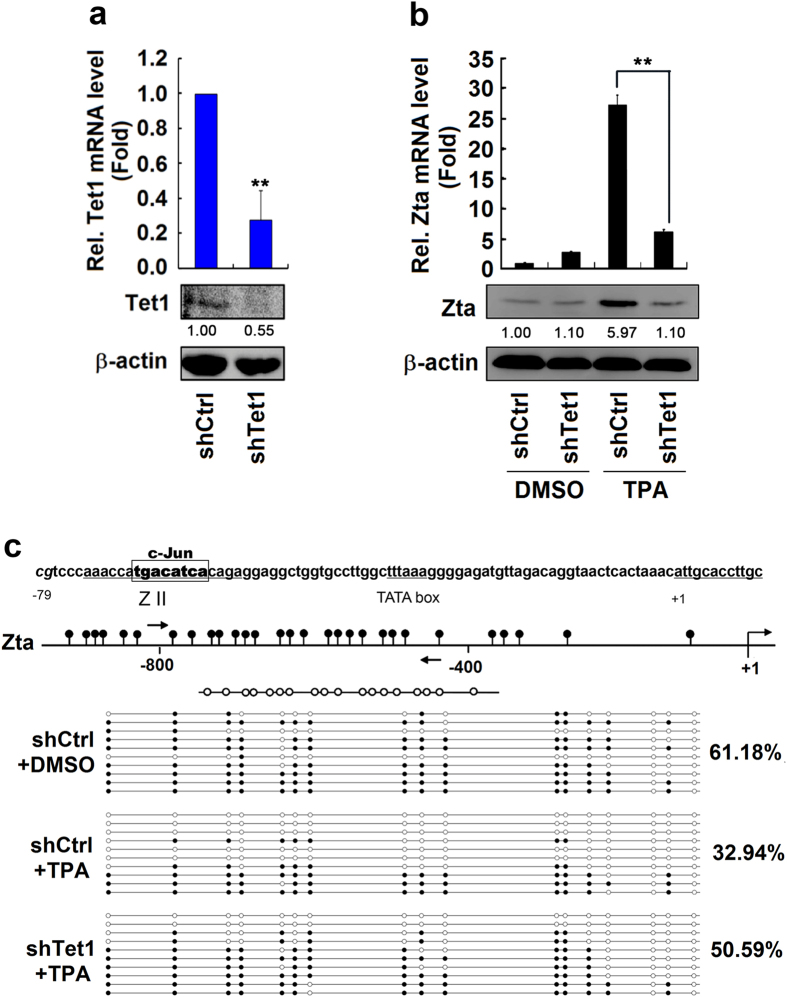
Tet1 is essential for TPA-mediated *Zta* promoter demethylation and gene expression. (**a**) B95-8 cells were transduced with lentiviruses expressing control shRNA (shCtrl) or shRNA against Tet1 (shTet1) for 2 weeks, and selected with puromycin. The total RNAs were isolated form the cells and the level of *Tet1* mRNA was determined by qRT-PCR (upper panel). The cell extracts were prepared and the level of Tet1 and β-actin proteins were detected by Western blot analyses (lower panel). (**b**,**c**) B95-8 cells were transduced with lentiviruses expressing shCtrl or shTet1 for 2 weeks, selected with puromycin, and then treated with TPA for 18 h. The total RNAs were isolated form the cells and the level of *Zta* mRNA was determined by qRT-PCR (b, upper panel). The cell extracts were prepared and the level of Zta and β-actin proteins were detected by Western blot analyses (b, lower panel). The *Zta* gene promoter was analyzed by sodium bisulphite sequencing. White and black circles indicate unmethylated and methylated CpGs, respectively (**c**). All data were collected from 3 independent experiments. *P < 0.05, **P < 0.01 and ***P < 0.001.

The role of Tet1 in the activation of *Zta* gene mediated by c-Jun was further evaluated. *Tet1* mRNA and Tet1 protein were reduced by lentivirus expressed shRNA against Tet1 (shTet1) in B95-8 cells (Fig. 5a), indicating shTet1 is effective. B95-8 cells were then treated with TPA and transfected with shTet1. The results showed that *Zta* mRNA and Zta protein were stimulated by TPA, but TPA-mediated activations were significantly repressed by shTet1 (Fig. 5b). The requirement of Tet1 for ERK-dependent demethylation of *Zta* promoter was then determined. The results revealed that *Zta* promoter methylation was significantly repressed by TPA (61.18% *vs.* 32.94%), but TPA-mediated repression was recovered by shTet1 (32.94% *vs.* 50.59%) (Fig. 5c). These results demonstrated that Tet1 is required for ERK-dependent *Zta* promoter DNA demethylation and gene expression.

### c-Jun and Tet1 are essential for ERK-dependent reactivation of EBV

**Figure 6:**
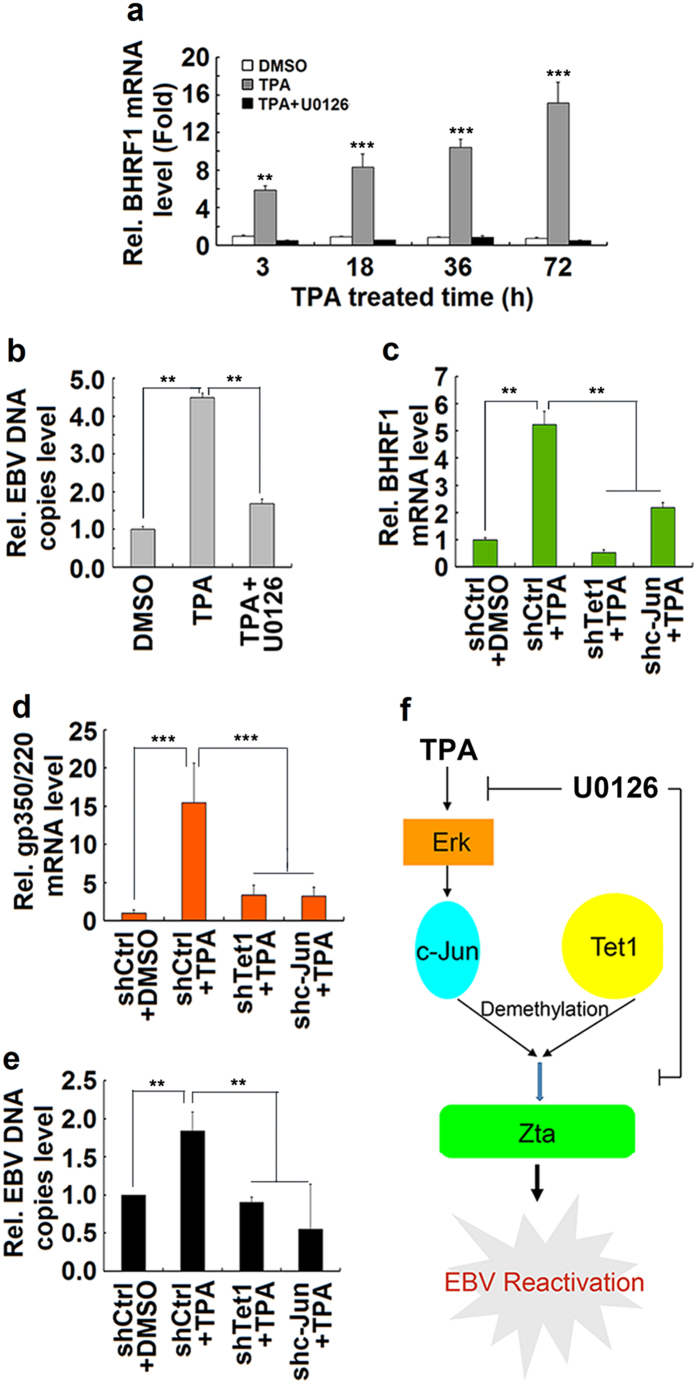
c-Jun and Tet1 are essential for ERK-dependent reactivation of EBV. **(a)** B95-8 cells were pretreated with U0126 (20 μM) for 2 h and then treated with TPA (60 ng/ml) for different times. RNA was extracted and the *BHRF1* gene mRNA was analyzed by qPCR. **(b)** B95-8 cells were pretreated with U0126 (20 μM) for 2 h and then treated with TPA (60 ng/ml) for 18 h. DNA was prepared from treated cells and the level of EBV genomic DNA was determined by qPCR. Values of viral genome copies are normalized to the control DMSO. (**c**–**e**) B95-8 cells were transduced with lentiviruses expressing shCtrl or shTet1 for 2 weeks, selected with puromycin, and then treated with TPA for 18 h. The total RNAs were isolated form the cells and the levels of *BHRF1* mRNA (**c**), *pg350/220* mRNA (**d**), and EBV genomic DNA (**e**) were determined by qPCR. Values are normalized to the control shCtrl. Error bars represent SD from the mean. All data were collected from 3–6 independent experiments. *P < 0.05, **P < 0.01 and ***P < 0.001.

The role of ERK pathway in the regulation of EBV reactivation was determined. To evaluate the effect of ERK pathway on the regulation of viral lytic gene expression, B95-8 cells were pretreated with U0126 and then treated with TPA for different times. BHRF1 (a key viral lytic early gene) mRNA was stimulated by TPA in a time-dependent manner, but TPA-mediated activation was repressed by U0126 (Fig. 6a). To determine the role of ERK in the regulation of EBV replication, B95-8 cells were pretreated with U0126 and then treated with TPA for 24 h. EBV genomic DNA copy number was activated by TPA in the absence of U0126, but not in the presence of U0126 (Fig. 6b), suggesting that ERK is required for the reactivation of EBV induced by TPA.

Furthermore, the effects of c-Jun and Tet1 on the expression of BHRF1 and gp350/220 (a key viral lytic late gene) and the replication of EBV were determined by using RNAi approach. B95-8 cells were transduced with lentiviruses expressing shCtrl, shc-Jun, and shTet1 for 2 weeks at puromycin selection and then treated with TPA for 18 h. *BHRF1* mRNA (Fig. 6c) and gp350/220 mRNA (Fig. 6d) were up-regulated by TPA, but such enhancements were down-regulated by shTet1 or shc-Jun, indicating that Tet1 and c-Jun are required for TPA-stimulated activation of viral lytic early gene and lytic late gene. Finally, EBV genomic DNA copy number was stimulated by TPA, but such activation was attenuated by shTet1 or shc-Jun (Fig. 6e), revealing that Tet1 and c-Jun are required for TPA-stimulated reactivation of EBV. Taken together, we demonstrated that Tet1 and c-Jun are required for ERK-dependent EBV gene expression and reactivation in response to TPA stimulation.

## Discussion

Methylation of CpG sites in the vicinity of the herpes virus immediate-early gene promoter usually inhibits gene expression and maintains viral latent infection^35^,^42^. In the contrary, DNA demethylation occurs during virus reactivation *in vivo* or after the treatment with phorbol ester TPA or DNA methyltransferase inhibitor 5-azacytidine *in vitro*^33^,^35^,^43^. However, the molecular mechanism underlying in the process of DNA demethylation is not understood. In this report, we document evidence that ERK signal pathway directs DNA demethylation stimulated by TPA. U0126, an ERK signaling pathway specific inhibitor, impedes *Zta* promoter demethylation. Ser73 phosphorylation of c-Jun, an ERK downstream transcription factor, is required for *Zta* gene transcription. Expectedly, depletion of c-Jun in B95-8 cells abrogates TPA-stimulated DNA demethylation of *Zta* promoter. More importantly, we demonstrate for the first time that c-Jun (an important transcription factor in the Activator protein-1 family) interacts with Tet1 (an important DNA modifying enzyme in the Ten-eleven translocation hydroxylase family) and recruits Tet1 to the *Zta* promoter region, leading to the initiation of *Zta* promoter DNA demethylation, IE gene expression, followed by lytic early and late gene expression, and ultimately EBV reactivation (Fig. 6f).

Previous studies have focused on *Zta* proximal promoter region (−221 to +12) and identified many important transcription factor binding elements essential for EBV latency or lytic infection, including c-Jun, ATF1, Sp1/3, MEF2D and ZEB1^15^,^18^,^19^,^37^,^44^. Additionally, there is only one CpG site in the proximal section and different sensitivity of cell types to DNA methyltransferase inhibitor^44^,^45^,^46^, and therefore, the significance of DNA demethylation on regulating *Zta* gene expression is always undervalued. In this study, we expand the bisulfite genomic sequencing region from −400 to −800 where is abundant with CpG sites. We further demonstrated that *Zta* gene promoter DNA methylation is significantly decreases in respond to TPA stimulation. The incomplete DNA methylation on *Zta* gene promoter implicates that the low spontaneous lytic level is permitted. Furthermore, Zta protein preferentially binds to and activates the cytosine-methylated silent viral promoters^30^,^47^,^48^,^49^,^50^,^51^, and also auto-regulates itself promoter^52^, so Zta may replace Tet1 to promote EBV reactivation without any more DNA demethylation. Therefore, we revel that TPA induces DNA demethylation, but TPA can not present a completely unmethylated status on Zta promoter.

c-Jun is a founding member of the activator protein-1 (AP-1) transcription factor family involved in cell proliferation, transformation, apoptosis, carcinogenesis^41^,^53^,^54^. In *p185*^BCR-ABL^-transformed cells, lack of c-Jun leads to methylation within the 5′ region of cell-cycle kinase 6 gene (*Cdk6*) and down-regulate the production of CDK6 protein^38^, which implicates that c-Jun may play a key role in maintaining hypomethylation of *Cdk6*. Recently, the DNA dioxygenase Tet1 has been reported to play an important role in the reprogramming of somatic cells to pluripotency^55^,^56^,^57^,^58^, and c-Jun impedes somatic cell reprogramming^40^. Combination with our finding that c-Jun interacts with Tet1 and recruits Tet1 to *Zta* gene promoter, it is possible that Tet1 is hijacked by c-Jun to regulate the expression of a subset of key reprogramming target genes. However, the assumption still needs further investigation.

In conclusion, we reveal that the activation of ERK/c-Jun signaling is required for the recruiting of Tet1 to the viral immediate-early gene promoter, which leads to the induction of DNA demethylation and viral reactivation. These results would provide new insights into our understanding the role of ERK signaling in the regulation of epigenetic modification changes and also would provide new insights into the mechanism underlying the regulation of EBV reactivation.

## Methods

For details of Methods, see Supplemental Methods.

### Plasmids, antibodies, and regents

The plasmid HA-Tet1 CD (pAAV-EF1a-HA-hTet1CD-WPRE-PolyA) was a gift from Hongjun Song (Addgene plasmid # 39454)^25^. We cloned the *c-Jun* cDNA (isolated from HEK293T cells) into pCMV-Tag 2B (Stratagene) and pGEX-6p-1 (GE Healthcare) vector to create Flag-c-Jun and GST-c-Jun, respectively.

Antibodies against EBV Zta (ZEBRA) (sc-53904, 1:200), c-Jun (sc-1694), c-Fos (sc-52) and ERK (sc-153) were purchased from Santa Cruz Technology Company. Rabbit polyclonal antibodies against p-ERK (p-P44/42 MAPK, *#* 4370S), p-c-Jun (Ser73, *#* 3270P) and p-c-Fos (Ser32, *#* 5348S) were obtained from Cell Signaling Technology. Mouse monoclonal anti-Flag antibody (Cat*#* F1804) and anti-HA antibody (Cat*#* H6908) produced in rabbit were purchased from Sigma Aldrich. Polyclonal antibody against Tet1 (Cat*#* GTX124207) was purchased from Gene Tex. Antibodies against 5-methylcytosine (5mC, MABE146) and 5-Hydroxymethylcytidine (5hmC, Cat*#* 39769) were obtained from Millipore and Active Motif, respectively.

The TPA (12-O-Tetradecanoylphorbol-13-acetate) (Cat*#* P8139) and U0126 (Cat*#* U120) were purchased from Sigma Aldrich.

## Additional Information

**How to cite this article**: Zhang, W. *et al.* ERK/c-Jun Recruits Tet1 to Induce *Zta* Expression and Epstein-Barr Virus Reactivation through DNA Demethylation. *Sci. Rep.* **6**, 34543; doi: 10.1038/srep34543 (2016).

## Supplementary Material

Supplementary Information

## References

[b1] Tse E. & Kwong Y. L. Epstein Barr virus-associated lymphoproliferative diseases: the virus as a therapeutic target. Exp Mol Med 47, e136 (2015). 25613733 10.1038/emm.2014.102 PMC4314579

[b2] Li L. *et al.* Methylation profiling of Epstein-Barr virus immediate-early gene promoters, BZLF1 and BRLF1 in tumors of epithelial, NK- and B-cell origins. BMC Cancer 12, 125 (2012). 22458933 10.1186/1471-2407-12-125 PMC3362778

[b3] Salvetti M., Giovannoni G. & Aloisi F. Epstein-Barr virus and multiple sclerosis. Curr Opin Neurol 22, 201–206 (2009). 19359987 10.1097/WCO.0b013e32832b4c8d

[b4] Young L. S. & Rickinson A. B. Epstein-Barr virus: 40 years on. Nat Rev Cancer 4, 757–768 (2004). 15510157 10.1038/nrc1452

[b5] Epstein M. A., Achong B. G. & Barr Y. M. Virus Particles in Cultured Lymphoblasts from Burkitt’s Lymphoma. Lancet 1, 702–703 (1964). 14107961 10.1016/s0140-6736(64)91524-7

[b6] Miyashita E. M., Yang B., Lam K. M., Crawford D. H. & Thorley-Lawson D. A. A novel form of Epstein-Barr virus latency in normal B cells *in vivo*. Cell 80, 593–601 (1995). 7532548 10.1016/0092-8674(95)90513-8

[b7] Chevallier-Greco A. *et al.* Both Epstein-Barr virus (EBV)-encoded trans-acting factors, EB1 and EB2, are required to activate transcription from an EBV early promoter. EMBO J 5, 3243–3249 (1986). 3028777 10.1002/j.1460-2075.1986.tb04635.x PMC1167318

[b8] Miller G. The switch between EBV latency and replication. Yale J Biol Med 62, 205–213 (1989). 2549738 PMC2589219

[b9] Yu K. P. *et al.* Latency of Epstein-Barr virus is disrupted by gain-of-function mutant cellular AP-1 proteins that preferentially bind methylated DNA. Proc Natl Acad Sci USA 110, 8176–8181 (2013). 23625009 10.1073/pnas.1301577110 PMC3657822

[b10] Tsurumi T., Fujita M. & Kudoh A. Latent and lytic Epstein-Barr virus replication strategies. Rev Med Virol 15, 3–15 (2005). 15386591 10.1002/rmv.441

[b11] Kenney S. C. & Mertz J. E. Regulation of the latent-lytic switch in Epstein-Barr virus. Semin Cancer Biol 26, 60–68 (2014). 24457012 10.1016/j.semcancer.2014.01.002 PMC4048781

[b12] Fenton M. & Sinclair A. J. Divergent requirements for the MAPK(ERK) signal transduction pathway during initial virus infection of quiescent primary B cells and disruption of Epstein-Barr virus latency by phorbol esters. J Virol 73, 8913–8916 (1999). 10482653 10.1128/jvi.73.10.8913-8916.1999 PMC112920

[b13] Fahmi H., Cochet C., Hmama Z., Opolon P. & Joab I. Transforming growth factor beta 1 stimulates expression of the Epstein-Barr virus BZLF1 immediate-early gene product ZEBRA by an indirect mechanism which requires the MAPK kinase pathway. J Virol 74, 5810–5818 (2000). 10846060 10.1128/jvi.74.13.5810-5818.2000 PMC112075

[b14] Liang C. L., Chen J. L., Hsu Y. P., Ou J. T. & Chang Y. S. Epstein-Barr virus BZLF1 gene is activated by transforming growth factor-beta through cooperativity of Smads and c-Jun/c-Fos proteins. J Biol Chem 277, 23345–23357 (2002). 11971895 10.1074/jbc.M107420200

[b15] Gao X., Ikuta K., Tajima M. & Sairenji T. 12-O-tetradecanoylphorbol-13-acetate induces Epstein-Barr virus reactivation via NF-kappaB and AP-1 as regulated by protein kinase C and mitogen-activated protein kinase. Virology 286, 91–99 (2001). 11448162 10.1006/viro.2001.0965

[b16] Borras A. M., Strominger J. L. & Speck S. H. Characterization of the ZI domains in the Epstein-Barr virus BZLF1 gene promoter: role in phorbol ester induction. J Virol 70, 3894–3901 (1996). 8648726 10.1128/jvi.70.6.3894-3901.1996 PMC190267

[b17] Ruf I. K. & Rawlins D. R. Identification and characterization of ZIIBC, a complex formed by cellular factors and the ZII site of the Epstein-Barr virus BZLF1 promoter. J Virol 69, 7648–7657 (1995). 7494273 10.1128/jvi.69.12.7648-7657.1995 PMC189705

[b18] Flemington E. & Speck S. H. Identification of phorbol ester response elements in the promoter of Epstein-Barr virus putative lytic switch gene BZLF1. J Virol 64, 1217–1226 (1990). 2154605 10.1128/jvi.64.3.1217-1226.1990 PMC249236

[b19] Feng W. H. *et al.* ZEB1 and c-Jun levels contribute to the establishment of highly lytic Epstein-Barr virus infection in gastric AGS cells. J Virol 81, 10113–10122 (2007). 17626078 10.1128/JVI.00692-07 PMC2045427

[b20] Robertson K. D. DNA methylation and chromatin - unraveling the tangled web. Oncogene 21, 5361–5379 (2002). 12154399 10.1038/sj.onc.1205609

[b21] Fernandez A. F. *et al.* The dynamic DNA methylomes of double-stranded DNA viruses associated with human cancer. Genome Res 19, 438–451 (2009). 19208682 10.1101/gr.083550.108 PMC2661803

[b22] Lister R. *et al.* Human DNA methylomes at base resolution show widespread epigenomic differences. Nature 462, 315–322 (2009). 19829295 10.1038/nature08514 PMC2857523

[b23] Bestor T. H. The DNA methyltransferases of mammals. Hum Mol Genet 9, 2395–2402 (2000). 11005794 10.1093/hmg/9.16.2395

[b24] Tahiliani M. *et al.* Conversion of 5-methylcytosine to 5-hydroxymethylcytosine in mammalian DNA by MLL partner TET1. Science 324, 930–935 (2009). 19372391 10.1126/science.1170116 PMC2715015

[b25] Guo J. U., Su Y., Zhong C., Ming G. L. & Song H. Hydroxylation of 5-methylcytosine by TET1 promotes active DNA demethylation in the adult brain. Cell 145, 423–434 (2011). 21496894 10.1016/j.cell.2011.03.022 PMC3088758

[b26] Hu L. *et al.* Structural insight into substrate preference for TET-mediated oxidation. Nature 527, 118–122 (2015). 26524525 10.1038/nature15713

[b27] He Y. F. *et al.* Tet-mediated formation of 5-carboxylcytosine and its excision by TDG in mammalian DNA. Science 333, 1303–1307 (2011). 21817016 10.1126/science.1210944 PMC3462231

[b28] Ito S. *et al.* Tet proteins can convert 5-methylcytosine to 5-formylcytosine and 5-carboxylcytosine. Science 333, 1300–1303 (2011). 21778364 10.1126/science.1210597 PMC3495246

[b29] Cortellino S. *et al.* Thymine DNA glycosylase is essential for active DNA demethylation by linked deamination-base excision repair. Cell 146, 67–79 (2011). 21722948 10.1016/j.cell.2011.06.020 PMC3230223

[b30] Woellmer A., Arteaga-Salas J. M. & Hammerschmidt W. BZLF1 governs CpG-methylated chromatin of Epstein-Barr Virus reversing epigenetic repression. PLoS Pathog 8, e1002902 (2012). 22969425 10.1371/journal.ppat.1002902 PMC3435241

[b31] Robertson K. D. & Ambinder R. F. Methylation of the Epstein-Barr virus genome in normal lymphocytes. Blood 90, 4480–4484 (1997). 9373258

[b32] Paulson E. J. & Speck S. H. Differential methylation of Epstein-Barr virus latency promoters facilitates viral persistence in healthy seropositive individuals. J Virol 73, 9959–9968 (1999). 10559309 10.1128/jvi.73.12.9959-9968.1999 PMC113046

[b33] Chan A. T. *et al.* Azacitidine induces demethylation of the Epstein-Barr virus genome in tumors. J Clin Oncol 22, 1373–1381 (2004). 15007085 10.1200/JCO.2004.04.185

[b34] Ben-Sasson S. A. & Klein G. Activation of the Epstein-Barr virus genome by 5-aza-cytidine in latently infected human lymphoid lines. Int J Cancer 28, 131–135 (1981). 6172387 10.1002/ijc.2910280204

[b35] Chen J. *et al.* Activation of latent Kaposi’s sarcoma-associated herpesvirus by demethylation of the promoter of the lytic transactivator. Proc Natl Acad Sci USA 98, 4119–4124 (2001). 11274437 10.1073/pnas.051004198 PMC31189

[b36] Falk K. I. & Ernberg I. Demethylation of the Epstein-barr virus origin of lytic replication and of the immediate early gene BZLF1 is DNA replication independent. Brief report. Arch Virol 144, 2219–2227 (1999). 10603176 10.1007/s007050050636

[b37] Wang Y. C., Huang J. M. & Montalvo E. A. Characterization of proteins binding to the ZII element in the Epstein-Barr virus BZLF1 promoter: transactivation by ATF1. Virology 227, 323–330 (1997). 9018131 10.1006/viro.1996.8326

[b38] Kollmann K. *et al.* c-JUN promotes BCR-ABL-induced lymphoid leukemia by inhibiting methylation of the 5′ region of Cdk6. Blood 117, 4065–4075 (2011). 21300982 10.1182/blood-2010-07-299644

[b39] Lake R. J. *et al.* The Sequence-Specific Transcription Factor c-Jun Targets Cockayne Syndrome Protein B to Regulate Transcription and Chromatin Structure. Plos Genetics 10 (2014). 10.1371/journal.pgen.1004284 PMC3990521 24743307

[b40] Liu J. *et al.* The oncogene c-Jun impedes somatic cell reprogramming. Nat Cell Biol 17, 856–867 (2015). 26098572 10.1038/ncb3193

[b41] Shaulian E. & Karin M. AP-1 as a regulator of cell life and death. Nature Cell Biology 4, E131–E136 (2002). 11988758 10.1038/ncb0502-e131

[b42] Robertson K. D. The role of DNA methylation in modulating Epstein-Barr virus gene expression. Curr Top Microbiol Immunol 249, 21–34 (2000). 10802936 10.1007/978-3-642-59696-4_2

[b43] Yang Z., Tang H., Huang H. & Deng H. RTA promoter demethylation and histone acetylation regulation of murine gammaherpesvirus 68 reactivation. PLoS One 4, e4556 (2009). 19234612 10.1371/journal.pone.0004556 PMC2644783

[b44] Speck S. H. & Ganem D. Viral latency and its regulation: lessons from the gamma-herpesviruses. Cell Host Microbe 8, 100–115 (2010). 20638646 10.1016/j.chom.2010.06.014 PMC2914632

[b45] Countryman J. K., Gradoville L. & Miller G. Histone hyperacetylation occurs on promoters of lytic cycle regulatory genes in Epstein-Barr virus-infected cell lines which are refractory to disruption of latency by histone deacetylase inhibitors. J Virol 82, 4706–4719 (2008). 18337569 10.1128/JVI.00116-08 PMC2346723

[b46] Murata T. & Tsurumi T. Epigenetic modification of the Epstein-Barr virus BZLF1 promoter regulates viral reactivation from latency. Front Genet 4, 53 (2013). 23577022 10.3389/fgene.2013.00053 PMC3620531

[b47] Bhende P. M., Seaman W. T., Delecluse H. J. & Kenney S. C. The EBV lytic switch protein, Z, preferentially binds to and activates the methylated viral genome. Nat Genet 36, 1099–1104 (2004). 15361873 10.1038/ng1424

[b48] Bergbauer M. *et al.* CpG-methylation regulates a class of Epstein-Barr virus promoters. PLoS Pathog 6, e1001114 (2010). 20886097 10.1371/journal.ppat.1001114 PMC2944802

[b49] Dickerson S. J. *et al.* Methylation-dependent binding of the epstein-barr virus BZLF1 protein to viral promoters. PLoS Pathog 5, e1000356 (2009). 19325883 10.1371/journal.ppat.1000356 PMC2654727

[b50] Karlsson Q. H., Schelcher C., Verrall E., Petosa C. & Sinclair A. J. Methylated DNA recognition during the reversal of epigenetic silencing is regulated by cysteine and serine residues in the Epstein-Barr virus lytic switch protein. PLoS Pathog 4, e1000005 (2008). 18369464 10.1371/journal.ppat.1000005 PMC2267006

[b51] Kalla M., Schmeinck A., Bergbauer M., Pich D. & Hammerschmidt W. AP-1 homolog BZLF1 of Epstein-Barr virus has two essential functions dependent on the epigenetic state of the viral genome. Proc Natl Acad Sci USA 107, 850–855 (2010). 20080764 10.1073/pnas.0911948107 PMC2818922

[b52] Taylor N. *et al.* ZEBRA and a Fos-GCN4 chimeric protein differ in their DNA-binding specificities for sites in the Epstein-Barr virus BZLF1 promoter. J Virol 65, 4033–4041 (1991). 1649314 10.1128/jvi.65.8.4033-4041.1991 PMC248834

[b53] Eferl R. & Wagner E. F. AP-1: a double-edged sword in tumorigenesis. Nat Rev Cancer 3, 859–868 (2003). 14668816 10.1038/nrc1209

[b54] Ozanne B. W., Spence H. J., McGarry L. C. & Hennigan R. F. Transcription factors control invasion: AP-1 the first among equals. Oncogene 26, 1–10 (2007). 16799638 10.1038/sj.onc.1209759

[b55] Chen J. *et al.* Vitamin C modulates TET1 function during somatic cell reprogramming. Nat Genet 45, 1504–1509 (2013). 24162740 10.1038/ng.2807

[b56] Costa Y. *et al.* NANOG-dependent function of TET1 and TET2 in establishment of pluripotency. Nature 495, 370–374 (2013). 23395962 10.1038/nature11925 PMC3606645

[b57] Gao Y. *et al.* Replacement of Oct4 by Tet1 during iPSC induction reveals an important role of DNA methylation and hydroxymethylation in reprogramming. Cell Stem Cell 12, 453–469 (2013). 23499384 10.1016/j.stem.2013.02.005

[b58] Chen J. *et al.* The combination of Tet1 with Oct4 generates high-quality mouse-induced pluripotent stem cells. Stem Cells 33, 686–698 (2015). 25331067 10.1002/stem.1879

