# Supplementary material for: ERK/c-Jun Recruits Tet1 to Induce Zta Expression and Epstein-Barr Virus Reactivation through DNA Demethylation
Source: Sci Rep. 2016 Oct 6;6:34543. doi: 10.1038/srep34543 (PMC5052586; doi:10.1038/srep34543)
Supplement: Supplementary Information [file srep34543-s1.doc]

**ERK/c-Jun Recruits Tet1 to induce *Zta* Expression and Epstein-Barr Virus Reactivation through DNA Demethylation**

Wei Zhang, Dongjie Han, Pin Wan, Pan Pan, Yanhua Cao, Yingle Liu**, Kailang Wu**, and Jianguo Wu*

State Key Laboratory of Virology and College of Life Sciences, Wuhan University, Wuhan 430072, China

**Supplemental Information**

**Supplemental Methods**

**Cell culture**

The EBV-positive and latent infection cell lines B95-8 with a low spontaneous virus production were grown in RPMI 1640 supplemented with 10% fetal bovine serum (GIBCO BRL, Grand Island, NY, USA) and 1% penicillin-streptomycin. For induction of the EBV lytic cycle, B95-8 cells were stimulated with 60 ng/ml of TPA. HEK293T cells were maintained in Dulbecco modified Eagle medium (DMEM) (GIBCO BRL) containing 10% fetal bovine serum (FBS). The cells were cultured at 37^o^C in a humidified atmosphere of 5% CO_2_.

**shRNA and lentivirus**

Tet1 shRNA (RNAi sequence: GCAAATCAACAGGAAGTTTCT) and c-Jun shRNA (RNAi sequence: CCTCAGCAACTTCAACCCA) were generated by insertion of the hairpin target sequence into the pGMLV-SC5 vector (Genomeditech Co. Ltd) double digested with *Bam*HI and *Eco*RI, and ligation with T4 DNA ligase in accordance with the manufacturer's guidelines. The ligations were transformed into competent *Escherichia coli* DH5α cells. The correct transformants were identified by DNA sequencing. HEK293T cells were transfected with expression lentivirus plasmid (shc-Jun or shTet1) together with package vectors by Lipo2000. 48 h after transfection, the medium containing the lentiviruses was collected and filtered concentrated.

For lentivirus infection, B95-8 cells were seeded in 6-well plates and cultured overnight. The lentiviruses were diluted in 1 ml complete medium and treated for 48 h at 37^o^C, then replaced with fresh culture medium containing 2 µg/ml puromycin for two weeks selection.

**Western blots**

Cells were harvested, washed briefly with ice-cold 1 x PBS and collected, and the pellets were suspended in WB lysis buffer (20 mM Hepes, 150 mM NaCl, 1% Triton X-100, 1 mM EDTA, 1 mM EGTA, 1% cocktail protease and phosphatase inhibitor, pH7.5) and sonicated. Lysates were clarified by centrifugation for 10 min at 10,000g. The protein concentration in each sample was determined by using the Bradford assay kit (Bio-Rad, Hercules, CA). Equal amounts of cultured cell lysates (80 μg) in loading buffer, heated for 5 min at 100°C and separated by SDS-PAGE on 10% gels and transferred to a nitrocellulose membrane (Amersham, Milwaukee, WI, USA). Nonspecific sites on membranes were blocked for 1 hour at room temperature in 5% nonfat dry milk in a 1 x PBS/0.1% Tween 20 (PBST) before being incubated with specific primary and secondary antibodies. Blots were analyzed by using a Luminescent Image Analyzer (Fujifilm LAS-4000, Tokyo, Japan).

**RNA extraction and real-time PCR**

Total RNA was extracted from cells by using TRIzol reagent following the manufacturer's instructions (Invitrogen). Total RNA extract was treated with DNase I (Promega, Madison, WI, USA) at 37°C for 30 min. Total RNA was used as a template for reverse transcription by M-MLV Reverse Transcriptase (Promega) using Oligo(dT)_15_ primer at 42°C for 90 min. Real-time PCR was performed using SYBR Green PCR master mix in a Light Cycler 480 thermal cycler (Roche, Risch-Rotkreuz, Switzerland) under the following conditions: heat activation of the polymerase at 95°C for 5 min, followed by 45 cycles of 95°C for 15 sec, 60°C for 15 sec, and 72°C for 20 sec. The fluorescence was then measured with a final melting curve step from 50°C to 95°C to assess the quality of the detection primers. Quantitative real-time PCR was performed with the following primer pairs: for Zta (BZLF1), forward 5′-AATGCCGGGCCAAGTTTAAGCAAC-3′ and reverse 5′-TTGGGCACATCTGCTTCAACAGGA-3′; for Tet1, forward 5′-CCCGAATCAAGCGGAAGA-3′ and reverse 5′-AGGAGAAGCCTGGAGATG-3′; for c-Jun, forward 5′-GTGCCGAAAAAGGAAGCTGG-3′ and reverse 5′-CTGCGTTAGCATGAGTTGGC-3′; for BMRF1, forward 5′- CTAGCCGTCCTGTCCAAGTGC-3′ and reverse 5′-AGCCAAACAGCTCCTTGCCCA-3′; for gp350/220, forward 5′-GTCAGTACACCATCCAGAGCC-3′ and reverse 5′-TTGGTAGACAGCCTTCGTATG-3′; for BHRF1, forward 5′- GTCAAGGTTTCGTCTGTGTG-3′ and reverse 5′-TTCTCTTGCTGCTAGCTCCA-3′; for internal reference GAPDH, forward 5′-GGAAGGTGAAGGTCGGAGTCAACGG-3′ and reverse 5′-CTCGCTCCTGGAAGATGGTGATGGG-3′. The results were normalized to GAPDH expression. All samples were analyzed in triplicate.

**Nucleic acid isolation and bisulfite genomic sequencing**

Genomic DNA was isolated from cells strictly according to the instructions provided by the manufacturer (Qiagen, Germany) and bisulfite converted using MethylCode™ Bisulfite Conversion Kit (Life). For bisulfite genomic sequencing, PCR primers were designed to anneal at both methylated and unmethylated bisulfite-converted DNA: pZta-BSP-F, AGYGATGAGAGATTTATATTTTTTTA and pZta-BSP-R, CAACCATCTCCCTTAAAACCTT. PCR products were gel excised and cloned directly into TA cloning vector, and sequencing using the M13 reverse primers.

For quantification of EBV DNA copy number, DNA was extracted from both cells and the supernatants using a QIAamp DNA Blood mini Kit (Qiagen, Germany). EBV DNA was quantified using specific primers (sense primer: TTCATCACCGTCGCTGACTC; antisense primer: GGACGAGGACCCTTCTACGG and probe: FAM-CCGAAGTGAAGGCCCTGGACCAACC-TRAMA) by comparison with a *Bam*HI W fragment of EBV plasmid-derived stand curve.

**Detection of 5mC and 5hmC by dot blots**

300 ng of isolated genomic DNA was used for the experiment. DNA was firstly denatured at 100°C for 5min in a 0.1 M sodium hydroxide solution and neutralized using 0.1 vol of 6.6 M ammonium acetate on ice. 5 µl of the denatured DNA was slowly manually spotted onto a hybond N+ membrane (Amersham) and dried in a hybridization oven at 80°C for 10 min. The DNA was then UV-crosslinked using a transilluminator for 120 sec and blocked overnight in blocking buffer (10% BSA, PBST/PBS + 0.1% Tween-20) at 4°C. The membrane was incubated with anti 5mC antibody (1:1000) or anti 5hmC antibody (1:2000) diluted in 10ml of blocking buffer for 4 h followed by three washes with PBST. Specific secondary antibody conjugated to a horseradish peroxidase enzyme (HRP) was incubated with the membrane for 45min followed by three washes with PBST. Blots were analyzed by using a Luminescent Image Analyzer (Fujifilm LAS-4000, Tokyo, Japan). The same blots were also staining with 0.2% methylene blue for 30min to confirm loading.

**Yeast two-hybrid assay**

*Saccharomyces cerevisiae* AH109, control vector pGADT7, pGADT7-T, pGADT7-Lam, pGBKT7 and pGBKT7-p53 were purchased from Clontech (Mountain View, CA). Plasmid pGBKT7-c-Jun containing the GAL4 DNA-binding domain and pGADT7-Tet1 CD containing the GAL4 DNA-activating domain were co-transformed into AH109. Transformed yeast cells containing both plasmids were first grown on synthetic dropout (SD) medium lacking two nutrients tryptophan, leucine (SD-minus Trp/Leu plates, DDO), then colonies were replica-plated onto SD-minus Trp/Leu/Ade/His plates (QDO) containing X-α-gal to check for the expression of reporter gene (blue colonies). No interactions were identified between BD-c-Jun and control empty vector AD-Vector or between the control empty vector BD-Vector and AD-Tet1 CD or BD-p53 and AD-Lam which served as a negative control. Positive interactions were identified between BD-c-Jun and AD-Tet1 CD, as well as BD-p53 and AD-T, which served as a positive control.

**Co-immunoprecipitation and** **GST pull-down assay**

Cells were transfected with the indicated plasmids for 36 h and then lysed in 1 ml IP Lysis Buffer (25 mM Tris-HCl, pH7.4, 150 mM NaCl, 1 mM EDTA, 5% glycerol, 1 X cocktail protease and phosphatase inhibitor). After sonicated 7 times (5s per time) and centrifuged, the supernatants were collected, pre-cleared with protein A/G (Santa Cruz Biotechnology, Santa Cruz, CA, USA) agarose beads and incubated with the indicated antibodies overnight at 4°C. 30 µl of protein A/G agarose beads were added to each tube and rotated for 2 h at 4°C. The beads were washed five times with IP Lysis Buffer and eluted with 1% SDS. The immunoprecipitates were analyzed by SDS-PAGE and western blotting with the appropriate antibodies.

For GST pull-down assay, recombinant proteins GST or GST-c-Jun were expressed in and purified from *E. coli* BL21. The purified protein was bound to Glutathione Sepharose 4B for 1 h at 4°C and then washed with lysis buffer (1 x PBS, 1% Triton X-100) three times, then incubated with recombinant HA-Tet1 (aa1418-2136) harvested from transfected HEK293T cells overnight at 4°C. The eluted proteins were detected by SDS-PAGE and western blotting with the indicated antibodies.

**Chromatin immunoprecipitation (ChIP)**

The ChIP assay was performed according to EZ ChIP (catalog no.17-371, Millipore) instruction with some modification. B95-8 cells were pretreated for 2 h with U0126 (20 µM) before the addition of TPA (60 ng/ml) or vehicle. 4 hours later, cells were crosslinked by 1% formaldehyde for 10 min and lysed in Lysis Buffer (10 mM Tris-HCl, pH8.0, 50 mM NaCl, 10 mM EDTA, 20% Sucrose, 1 X cocktail protease and phosphatase inhibitor) for 30 min. After adding equal amount of 2 X IP Buffer (200 mM Tris-HCl, pH8.0, 600 mM NaCl, 4% Triton X-100, 2 x cocktail protease and phosphatase inhibitor), lysates were sonicated on ice and debris were removed by centrifugation at 15000g for 10 min at 4°C. 1% of the supernatant was used as DNA input control. The remaining supernatant was diluted 10-fold with Dilution Buffer (1% Triton X-100, 2 mM EDTA, 20 mM Tris-HCl, pH8.0, and 150 mM NaCl) and incubated with antibody against c-Jun or Tet1 and rotated overnight at 4°C. Immunoprecipitated complexes were collected by protein A/G agarose beads. The pellets were washed once with low salt wash buffer (0.1% SDS, 1% Triton X-100, 2 mM EDTA, 20 mM Tris-HCl, 150 mM NaCl), high salt wash buffer (0.1% SDS, 1% Triton X-100, 2 mM EDTA, 20 mM Tris-HCl, 500 mM NaCl), and Tris-EDTA buffer and incubated at 65°C for 5 h to reverse the formaldehyde cross-link. DNA was purified using E.Z.N.A®. Cycle-Pure Kit (D6492, Omega). The amount of c-Jun and Tet1 bound to the Zta (BZLF1) promoter was determined by qPCR using Zta (BZLF1) promoter-specific primers (forward: ATGAGCCACAGGCATTGCTAATGTA and reverse: GTGGCCGGCAAGGTGCAATGTTTAG).

**Immunofluorescence**

HEK293T cells were grown on sterile cover slips and co-transfected with HA-Tet1 and Flag-c-Jun constructs a day before fixation. Cells were fixed and permeabilized with equal volume of MeOH and acetone for 20 min at 4°C, washed three times with PBS, and blocked with PBS containing 3% bovine serum albumin for 1 h at 37°C. The cells were incubated with the primary antibodies, anti-HA (1:100) and anti-Flag (1:100) (Sigma-Aldrich), diluted in 3% bovine serum albumin in PBS for 1 h at room temperature, followed by incubation with fluorescein isothiocyanate (FITC)-conjugated goat anti-rabbit secondary antibodies and Cy3-conjugated goat anti-mouse secondary antibodies (1:200) (Proteintech Group, Chicago, IL, USA) diluted in 3% bovine serum albumin in PBS for 45 min. Nuclei were stained with DAPI (Roche, Basel, Switzerland) for 5 min at 37°C, the cells images were acquired with a confocal laser microscopy (Fluoview FV1000, Olympus).

**Statistical analyses**

All experiments were reproducible and repeated at least three times with similar results. Parallel samples were analyzed for normal distribution using Kolmogorov-Smirnov tests. Abnormal values were eliminated using a follow-up Grubbs test. Levene’s test for equality of variances was performed, which provided information for Student’s *t*-tests to distinguish the equality of means. Means were illustrated using histograms with error bars representing the SD; a *P* value of <0.05 was considered statistically significant.

**Supplemental Figures and Legends**

**
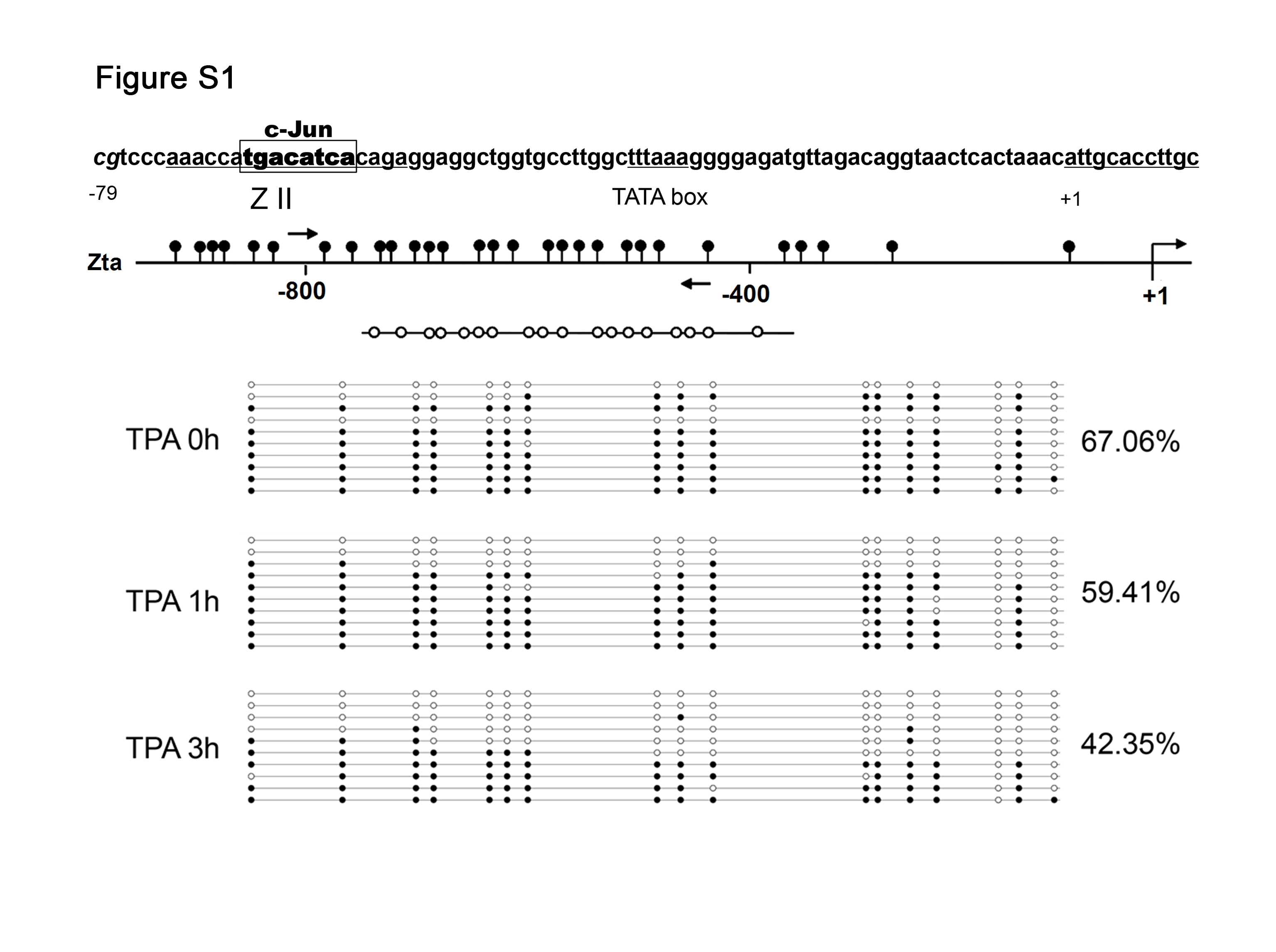
**

**Supplemental Figure S1. TPA stimulates DNA demethylation of the Zta promoter at early time.**

B95-8 cells were treated with TPA (phorbol ester 12-O-Tetradecanoylphorbol 13-acetate) (60 ng/ml) for the indicated time periods. The Zta promoter was analyzed by sodium bisulphite sequencing. The white and black circles indicate unmethylated and methylated CpGs, respectively.

**
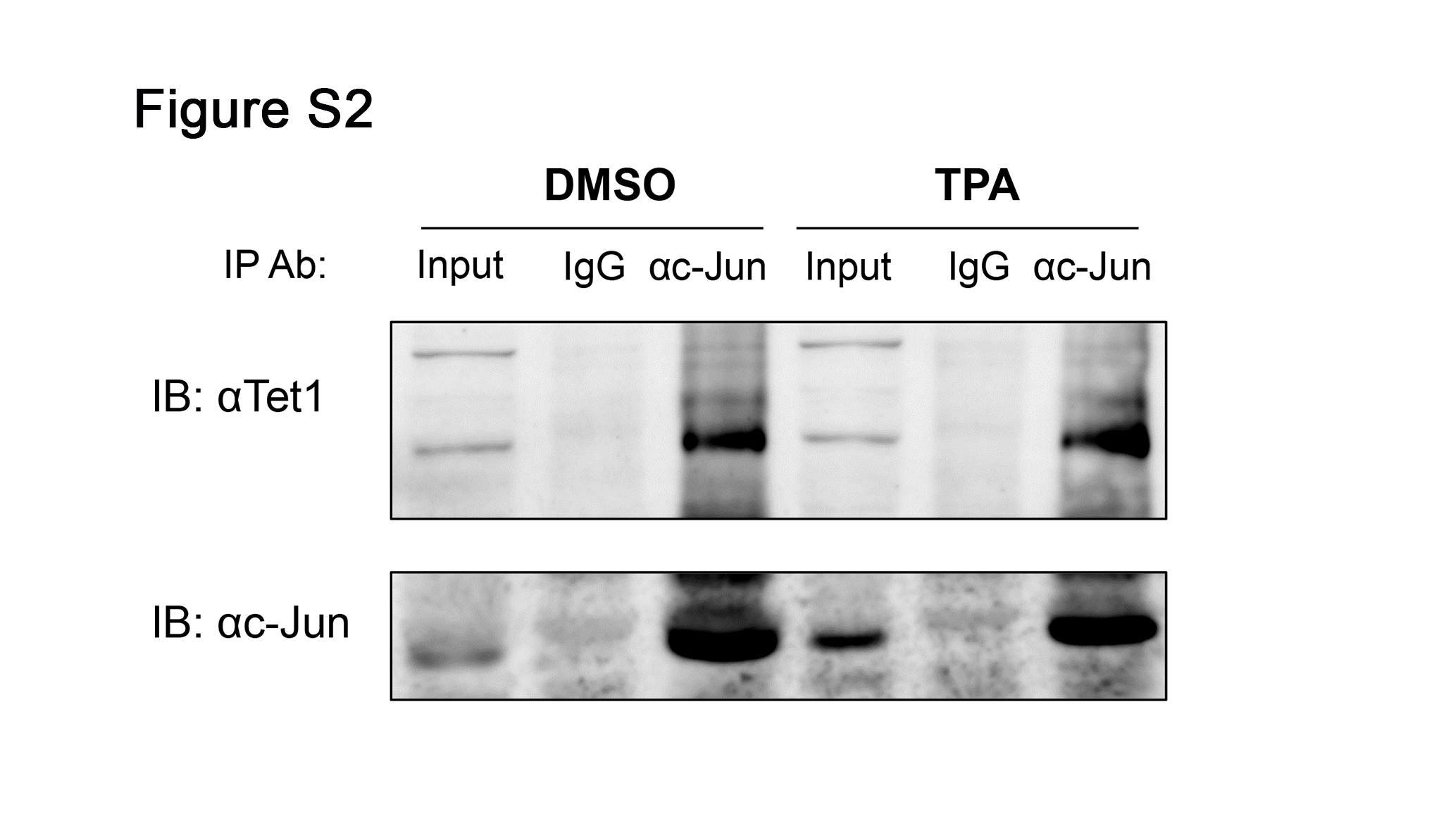
**

**Supplemental Figure S2. Coimmunoprecipitation of endogenous c-Jun and Tet1.**

B95-8 cells were treated with DMSO or TPA for 4 h, and whole-cell extracts were prepared. Tet1 was then immunoprecipitated and western blotting performed using the c-Jun antibody.
